# About Dinner Hour

**Published:** 1882-01

**Authors:** 


### ABOUT THE DINNER HOUR.

I GRANT that one o'clock may be the luncheon hour of that very small section of society which turns daylight into dark, and natural habits topsy-turvy; but can Mrs. Partington on tiptoe with her mop and pattens obliterate the course of the sun ? or Mrs. Grundy, with all Belgravia at her back, make fashionable morning out of four o'clock in the afternoon ? The silly attempt reminds me of a young French beau of the time of Louis XVIII., decked out in his court finery. He was just off to a grand ball, when his valet entered hastily with "Your mother is just dead !" "Impossible, my friend," replied the Count, looking at his pumps and vanities, "Madame cannot die until twelve o'clock to-night," and off he went to enjoy himself. The time for luncheon, moreover, is of very recent adoption, being about two hours after good Queen Bess had dined, pinched her maids and taken her afternoon nap. It may astonish some ladies who raise their hands and eyes at the bare possibility of dining by daylight to be told that the nobility and gentry of this realm during the middle ages dined at ten o'clock in the morning; and in the fourteenth century at eleven o'clock, sometimes being dissipated enough to sit "over the walnuts and the wine" until afternoon chapel at three. This was the division of the forenoon among the quality in the sixteenth century, when a wardrobe lasted longer than a life: "We wake at six, and look about us—that's eye hour; at seven we should pray—that's knee hour; at eight, walk—that's leg hour; at nine, gather flowers, and pluck a rose—that's nose hour; at ten we drink—that's mouth hour; at eleven, lay about us for victuals—that's hand hour; at twelve, go to dinner—that's belly hour." But the dinner hour soon became later. "The landmarks of our fathers," writes Steele in 1710, "are removed and planted further up on the day. In my own memory, the dinner hour has crept by degrees from twelve o'clock to three; where it will end, nobody knows." In the reign of

George II. the fashionable dinner hour was four o'clock; in that of the "Farmer King," six o'clock; and during the reign of that pattern of propriety, George IV., seven o'clock; and if we could get embalmed for two hundred years we should see it come back to ten in the morning. It is but fair to add that our continental neighbors have not yet overtaken us, for Ticknor mentions in 1835 that the King of Sweden dined at one, and I believe the Emperor of Germany still dines at five o'clock.

Thus, the custom of dining late is a mere mushroom of yesterday, and an uncalled-for innovation brought about by idle radicals (I use the term in a Pickwickian sense). The workingmen who dine at noon are the grand old conservatives. They adhere to the fashion of the Stuarts, and break their fast at the orthodox hour; and if we desire the gentlemen of England to take their meals as in the good old times, we have only to get the working men to dine at 8 o'clock in the evening, and then my Lord Bantam and my Lady Languish would soon right about face to the meridian meal. For who would think of sitting down to turbot and champagne at the very moment when Hodge was cutting three inches deep into the stale loaf and dividing a pound of bacon to meet six little untoward circumstances "with mouths like attic windows perpetually flying open?" The very fact that my neighbor dined at one would of course make me dine at two; just as I know a particularly sociable parish near Crawley where the lady who has two windows each side of the front door will not visit with the lady opposite because she has only one.—*Temple Bar.*

